# Routine physics work efficacy and efficiency improvement through a shared‐duty physicist‐of‐the‐day model in a multi‐hospital radiation oncology network

**DOI:** 10.1002/acm2.70792

**Published:** 2026-09-09

**Authors:** Guang‐Pei Chen, Douglas E. Prah, Eric S. Paulson

**Affiliations:** ^1^ Department of Radiation Oncology Medical College of Wisconsin Milwaukee Wisconsin USA

**Keywords:** multi‐site radiation oncology network, operational efficiency, patient safety, physicist‐of‐the‐day

## Abstract

**Background:**

Large multi‐hospital radiation oncology networks often rely on site‐exclusive physicist coverage models that can lead to imbalanced workload distribution, inconsistent task execution, communication fragmentation, and vulnerability to delayed or missed quality checks. Increasing clinical complexity and distributed staffing models necessitate operational strategies that improve efficiency, standardization, and safety across geographically separated sites.

**Purpose:**

To describe the implementation of a shared‐duty physicist‐of‐the‐day (POD) model supported by a real‐time operational dashboard and to evaluate its impact on workload distribution, task efficiency, and safety metrics across a multi‐hospital radiation oncology network.

**Methods:**

A structural transition from a site‐exclusive to a shared‐duty POD model was implemented in our institution—a four‐site academic radiation oncology system. A custom application, RadOncStatusBoard, was developed using structured query language (SQL)‐based integration with the Mosaiq Record & Verify system to aggregate real‐time patient appointments, treatment progress, quality checklist (QCL) items, and weekly chart check (WCC) eligibility. Microsoft Teams channels were used for daily communication and task coordination. Operational data from two matched 21‐month intervals—before and after POD restructuring—were extracted using SQL. Evaluated metrics included task volumes, claim times, completion times, WCC performance, billing accuracy, and dosimetry‐related Radiation Oncology Incident Learning System (RO‐ILS) event reporting. Right‐skewed distributions were summarized using medians and interquartile ranges (IQR). Surveys of physicists and dosimetrists were performed to assess end‐user perceptions of workflow and safety.

**Results:**

Workload balance improved across all task categories, with substantial decreases in main‐to‐satellite task ratios (e.g., the ratio for chart close decreased from 1.91 to 1.27). Task claim and completion times decreased markedly at both main and satellite locations (e.g., chart‐close claim time decreased from 36.8 to 6.2 min at the main campus). WCC completion improved from 97.7% to 99.9%, and WCC charge capture increased from 96.1% to 99.4%. The proportion of dosimetry‐related RO‐ILS events decreased numerically from 31.0% to 26.7% (*p* = 0.339). Survey responses from physicists and dosimetrists indicated improved workflow clarity, communication, workload balance, and perceived patient safety.

**Conclusions:**

Implementation of a shared‐duty POD model improved workload balance, enhanced operational efficiency, and strengthened routine safety processes across a multi‐site radiation oncology program. Integration of a real‐time operational dashboard, standardized communication pathways, and structured task‐claiming workflows enabled measurable gains in responsiveness, compliance, and perceived safety culture.

## 1 INTRODUCTION

Medical physicists play a central role in the safe and accurate delivery of radiation therapy by performing essential activities such as on‐screen plan reviews (OSPR), record‐and‐verify (R&V) transfer checks (RVTC), weekly chart checks (WCC), end‐of‐treatment chart review and closing (CC), image‐guided radiotherapy (IGRT) image review, special‐procedure support such as stereotactic body radiotherapy (SBRT), total‐body irradiation (TBI), orthovoltage, and troubleshooting of treatment‐planning or delivery issues. Most errors in radiotherapy originate during pretreatment,^1^ and initial physics plan checks (OSPRs and RVTCs), along with WCCs, are recognized as the first and second most effective quality‐control interventions, respectively.^2^ These activities ensure technical accuracy, detect deviations early, and maintain compliance with regulatory and accreditation standards.^3^, ^4^ Failure to complete routine tasks, such as missed WCCs, introduces clinical risk and jeopardizes quality metrics, as emphasized in incident‐learning analyses and national safety guidelines.^5^

Physics coverage models vary widely depending on institutional size, resources, geography, clinical volume, and jurisdiction‐specific legal and regulatory requirements. While single‐site dedicated coverage is common in small or medium centers,^6^ large multi‐hospital networks face unique operational challenges. Physicists are often assigned per satellite clinic or clinical service in siloed, satellite staff, or distributed effort models,^7^ mostly with routine responsibilities tied to a specific site. Variability in daily patient volume, treatment complexity, and staffing can create uneven workload distribution between main and satellite hospitals—a pattern described in prior assessments of distributed practice environments. Some institutions have introduced formal workload‐distribution frameworks, but these may not support real‐time task claiming or dashboard‐driven assignments.^8^ Although effective locally, site‐exclusive models can limit standardization, constrain responsiveness for urgent tasks, and contribute to uneven workload distribution.

Traditional WCC workflows—typically performed in batch mode on one or two designated weekdays for patients who have completed approximately one week of treatment—offer predictable scheduling but are vulnerable to patient‐schedule changes and fluctuations, BID treatments, atypical start days and emergency treatments. These factors increase the risk of delayed or missed checks, consistent with known limitations of batching‐based safety processes.^9^ Similarly, plan reviews performed predominantly by site‐specific physicists can increase variability in interpretation and workflow consistency, reflecting trends reported in human‐factors evaluations of physics quality assurance (QA) processes.^10^

To address these limitations, we implemented a shared‐duty physicist‐of‐the‐day (POD) model in which PODs across all clinical sites collaboratively manage non‐location‐dependent tasks through a centralized dashboard and real‐time communication platform. WCCs were transitioned to a rolling‐mode workflow—triggered by each patient's treatment fraction rather than a specific weekday. New patient starts at our institution were distributed throughout the workweek (29.63% Monday, 20.22% Tuesday, 20.33% Wednesday, 22.10% Thursday, and 7.55% Friday). Accordingly, this approach was designed to reduce workload clustering associated with fixed‐weekday WCC scheduling. Location‐dependent responsibilities, such as IGRT review, SBRT support, etc, remain site‐based. This approach aligns with professional recommendations and practice guidelines from the American Association of Physicists in Medicine (AAPM) and American Society for Radiation Oncology (ASTRO) emphasizing standardization, scalability, and quality improvement in physics workflows.^3^, ^11^, ^12^ Remote plan reviews and WCCs are feasible when supported by robust information technology infrastructure. Their effectiveness was demonstrated during the COVID‐19 pandemic,^13^, ^14^ and this practice is supported by ASTRO.^15^

The objective of this study was to (1) establish the workflow and necessary tools for implementing the restructured POD model, and (2) evaluate its impact on workload balance between main and satellite sites; efficiency of routine physics tasks; and safety metrics, including WCC performance, charge‐capture accuracy, and dosimetry‐related incident reporting.

## 2 METHODS AND MATERIALS

### 2.1 Clinical environment

Our radiation oncology program consists of one main academic center and three satellite clinics operating on a unified clinical platform. The network uses a beam‐matched treatment planning environment (Monaco, Elekta, Stockholm, Sweden; Precision, Accuray, Madison, Wisconsin) supported by a centralized R&V system (Mosaiq, Elekta). The main campus houses three Elekta c‐arm linacs (Infinity/VersaHD), one MR‐linac (Unity, Elekta), two helical tomotherapy systems (Radixact, Accuray), one pencil beam proton therapy system (S250i, Mevion, Littleton, Massachusetts), one HDR brachytherapy unit (Flexitron, Elekta), one orthovoltage unit (Sensus Healthcare), and one Gamma Knife radiosurgery system (Icon, Elekta). Each of the three satellite clinics operates one Elekta c‐arm linac.

Across the network, clinical radiation oncology services were supported by a department of 20 physicists and 10 dosimetrists. Although HDR, Gamma Knife, MR‐linac, and proton therapy procedures were covered by dedicated specialty physics teams, clinical physicists also rotated through the routine external beam POD schedule. Routine external beam services were provided by seven dosimetrists and three rotating physicist PODs working staggered shifts with overlapping coverage at the main campus, and by one dosimetrist and one rotating physicist POD at each satellite clinic. The POD responsibilities evaluated in this study focused on routine daytime clinical support and excluded scheduled patient‐ and machine‐specific quality assurance activities as well as emergency machine support. Core clinical responsibilities were largely consistent across sites, although the main campus also supported additional external beam procedures such as total body irradiation, motion management, and orthovoltage treatments. Historically, PODs operated in a site‐exclusive model, and WCCs were performed in a batch mode on Thursdays and Fridays by all available physicists rather than by the assigned POD team alone.

The Mosaiq quality checklist (QCL)^16^, ^17^ was implemented to track key dosimetry‐ and physics‐related tasks, including OSPRs (“PHY: On‐Screen Review*” and “FT PHY: On‐Screen Review*”), RVTCs (“PHY: Chart Check” and “FT PHY: Chart Check*”), CCs (“PHY: Chart Close*”), and other routine physics support activities. QCL items are either auto‐generated based on upstream task completion or manually created when additional support is required. Each item may be manually assigned a due date and due time to reflect the expected urgency of task completion. Under the site‐exclusive model, active claiming of OSPR QCLs was not enforced; instead, main‐campus dosimetrists assigned OSPRs through phone calls or office visits.

On October 16, 2023, the POD structure was transitioned to the shared‐duty model. Under this approach, all QCLs and WCCs were actively claimed by POD members, enabling real‐time task distribution and cross‐site workload sharing. Site‐specific physics support QCLs continued to be claimed by the POD assigned to the corresponding treatment location.

### 2.2 RadOncStatusBoard workflow integration

A custom software platform, RadOncStatusBoard, was developed to operationalize the shared‐duty POD workflow and provide a unified interface for real‐time monitoring of clinical physics activities. Implemented in Microsoft Visual C++ with Structured Query Language (SQL)‐based communication to the Mosaiq R&V system through open database connectivity (ODBC), the application integrates multiple data streams—including patient appointments, treatment progress, QCL items and WCC eligibility—into a centralized dashboard that supports coordinated POD operations across the cancer network.

RadOncStatusBoard retrieves daily patient treatment appointment data from Mosaiq and categorizes each encounter by real‐time status (Scheduled, Queued, Finished, No Show, Cancelled, On Hold, or Machine Down). These data are visualized for each treatment unit using bar‐chart or Gantt‐style timeline, enabling rapid identification of delays, cancellations, and anticipated machine completion times. For every patient scheduled for treatment, the application evaluates full‐course progress to determine WCC eligibility according to ASTRO recommendations. The eligibility algorithm accounts for BID treatments, schedule disruptions (including cancellations, no‐shows, machine downtime, and add‐on treatments), and seeks to avoid consecutive WCCs by the same physicist when possible. The current implementation does not consider whether the physicist who performed the pre‐treatment plan reviews (OSPR and RVTC) also performs the first WCC, because the OSPR and RVTC are completed by different PODs before the first WCC. The interface highlights WCC‐eligible patients and displays prior WCC and billing history, including flags for missed or incorrect charge capture.

The system also imports all uncompleted QCL tasks relevant to clinical physics activities, listing patient identifiers, task descriptions, QCL creation and due timestamps, and the responsible physicist. In parallel, unsigned physics documents, pending intensity‐modulated radiotherapy (IMRT) QA items, and other actionable tasks are listed for timely follow‐up. Item urgency is automatically color‐coded based on time‐to‐due, enabling effective prioritization.

All dashboard elements refresh automatically at predefined intervals, with an onscreen countdown indicating the next update. Users may also initiate a manual refresh at any time.

### 2.3 Microsoft teams operational framework

A Microsoft Teams “POD Teams” group was established with a new channel automatically created for each clinic day. The channel is used for POD updates, help seeking, issue escalation, coordination of urgent tasks, and documentation of daily operations. Teams‐based virtual handoffs have been shown to enhance clinical coordination in other domains.^18^

### 2.4 Data extraction and analysis

Operational and performance data were extracted from Mosaiq using SQL queries executed in Microsoft SQL Server Management Studio. Two matched 21‐month intervals were analyzed: the period before implementation of the shared‐duty POD model (1/16/2022–10/15/2023) and the period after implementation (10/16/2023–7/15/2025). For each interval, physics task assignments and QCL completion records were obtained, including task type, creation timestamp, claiming physicist, and completion timestamp. Workload balance was assessed by comparing the average number of QCLs completed by PODs at satellite clinics versus the main campus. Responsivness and efficiency were evaluated by examining task claim and completion times across both settings. Task claim time was defined as the interval from task creation to task claim, and task completion time was defined as the interval from task claim to task completion. Task claim times and task completion times were examined for distributional characteristics as part of the exploratory data analysis. Both variables exhibited right‐skewed distributions, with most observations having relatively short durations and a small number exhibiting substantially longer times. Therefore, results were summarized using medians and interquartile ranges (IQRs; 25^th^‐75^th^ percentiles), which provide robust measures of central tendency and variability.

Patients treated during the study windows were analyzed for adherence to ASTRO WCC guidelines. Using a custom SQL script, treatment delivery records, WCC dates, and associated billing data were retrieved. WCC performance was calculated as the proportion of required WCCs that were completed. Billing accuracy was quantified by identifying two categories of error: missed WCC charges (eligible but not billed) and inappropriate charges (billed despite ineligibility). This study was approved by the Institutional Review Board (IRB) under Approval No. PRO00022316 and was conducted in accordance with institutional ethical standards.

To evaluate the impact of POD restructuring on safety culture, we analyzed event reports from the ASTRO/AAPM Radiation Oncology Incident Learning System (RO‐ILS).^19^ Our department has participated in RO‐ILS since 2014, and all reported radiation oncology events—including scheduling, simulation, treatment planning, and treatment delivery events—are entered into RO‐ILS. All events from the two study intervals were reviewed, and dosimetry‐related incidents were classified according to RO‐ILS taxonomy. Total and dosimetry‐specific reporting proportions were compared using a two‐proportion z‐test, with *p* < 0.05 considered statistically significant. Because institutional incentive programs affected overall reporting volume, proportional metrics were used to mitigate reporting‐rate bias.

### 2.5 Survey administration

Two role‐specific surveys were administered to assess end‐user perceptions of the POD‐based physicist workflow: one distributed to clinical physicists and the other to dosimetrists. Surveys were conducted approximately two years after implementation to ensure respondents had sufficient experience with the restructured model. Items evaluated communication quality, task handoffs, workflow efficiency, prioritization of urgent work, resource adequacy, and overall satisfaction. Responses were collected using Likert‐scale categories (Strongly Agree to Strongly Disagree or equivalent), and descriptive statistics (frequencies and percentages) were calculated for each item. Participation was voluntary, and no identifying information was collected.

## 3 RESULTS

### 3.1 RadOncStatusBoard

**FIGURE 1:**
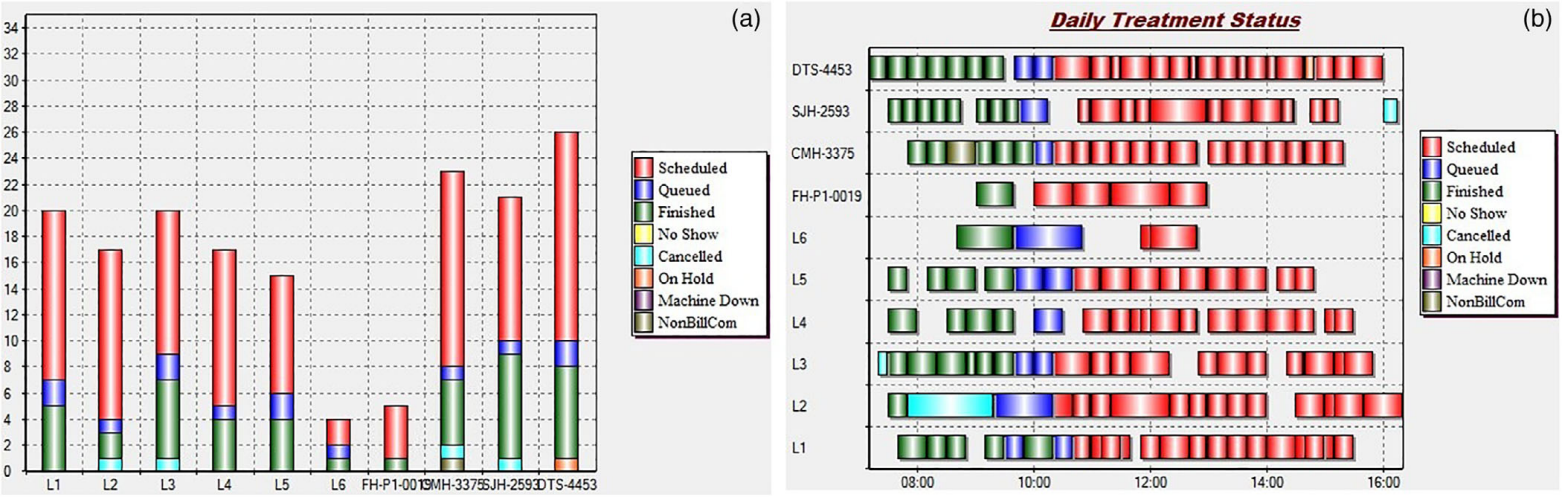
Patient treatment status displayed in RadOncStatusBoard using stacked‐column (A) and Gantt‐chart (B) viewing modes.

**FIGURE 2:**
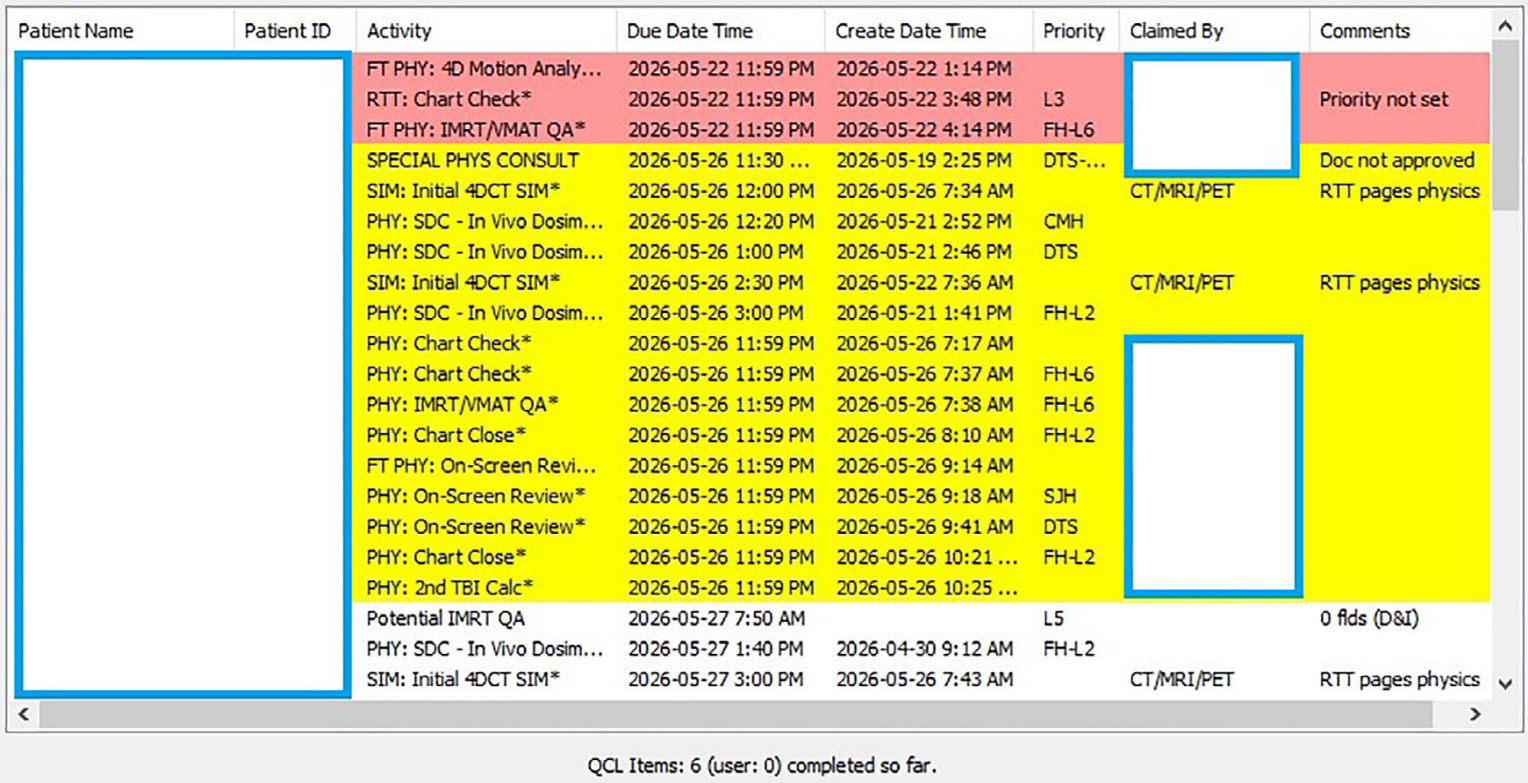
Mosaiq quality checklist (QCL) status displayed in RadOncStatusBoard. Unclaimed and claimed items due within 30 minutes are highlighted in red (none shown in this figure) and pink, respectively. Items due on the current day are highlighted in yellow.

**FIGURE 3:**
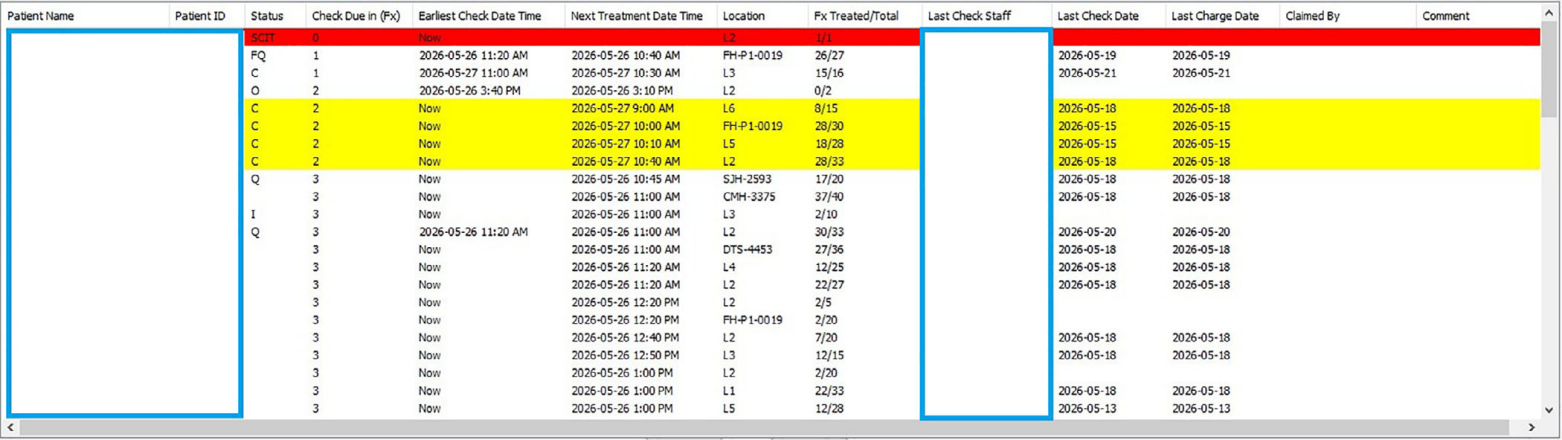
Patient WCC status displayed in RadOncStatusBoard. Patients highlighted in red have completed all treatments and require WCC completion on the current day. Patients highlighted in yellow are eligible for physicist review.

The RadOncStatusBoard was fully implemented and integrated into routine operations, serving as the central platform for monitoring physics tasks. After deployment, task visibility improved substantially, with real‐time updates allowing POD members to monitor treatment progress, identify outstanding QCLs and WCCs at a glance (Figures 1, 2, 3). The dashboard enabled automated flagging of overdue tasks and corrective items. No major technical issues were reported, and end‐user feedback indicated high usability and strong support for continued use.

### 3.2 Workload balance

The structural change was associated with clear improvements in workload balance between the main and satellite hospitals (Table 1). Before the intervention, the average number of QCL tasks completed per POD was consistently higher at the main campus across all categories. After restructuring, the main‐to‐satellite task ratios decreased for every task type, indicating a more equitable distribution of responsibilities.

**TABLE 1:** Comparison of the average number of tasks completed per POD at the main and satellite hospitals before and after the structural change, including the ratio of average tasks completed at the main versus satellite hospitals.

	Before			After		
Task	Main	Satellite	Main/ Satellite	Main	Satellite	Main/ Satellite
OSPR	2.86	1.19	2.41	2.13	1.62	1.31
RVTC	2.61	1.18	2.20	2.02	1.59	1.28
CC	1.97	1.03	1.91	1.75	1.38	1.27
Support	0.54	0.09	6.20	0.47	0.13	3.56

For CC tasks, the ratio decreased from 1.91 to 1.27. Similar reductions were observed for RVTCs (2.20 to 1.28) and OSPRs (2.41 to 1.31). The greatest shift occurred in support tasks, where the ratio fell from 6.20 to 3.56, reflecting substantial redistribution of support activities toward the satellite PODs. Overall, task‐volume disparities between sites were reduced across all routine clinical categories.

### 3.3 Responsivness and efficiency gains

- Task‐claim times

Task claim times decreased substantially following implementation of the shared‐duty model (Table 2). For CC tasks, median claim times dropped from 36.78 and 36.06 minutes at the main and satellite hospitals, respectively, before the change to 6.24 and 5.16 minutes after the change—an approximately sixfold improvement at both locations, reflecting substantially faster claiming of tasks. In addition to lower median claim times, the IQR decreased substantially following implementation, indicating that the middle 50% of task claim times became more tightly clustered.

**TABLE 2:** The median, and IQR of task claim time at the main and satellite hospitals, with corresponding main‐to‐satellite ratios.

	Before			After		
Task	Main (minutes)	Satellite (minutes)	Ratio	Main (Minutes)	Satellite (Minutes)	Ratio
OSPR	N/A	N/A	N/A	9.22/18.82	7.29/14.44	1.26/1.30
RVTC	N/A	N/A	N/A	7.17/13.51	6.42/11.86	1.12/1.14
CC	36.78/72.60	36.06/72.49	1.02/1.00	6.24/11.57	5.16/9.43	1.21/1.23

For OSPRs and RVTCs—tasks that were not formally claimed prior to restructuring—post‐implementation claim times were modestly longer at the main campus than at the satellites. Median claim times for OSPRs were 9.22 versus 7.29 minutes, and for RVTCs were 7.17 versus 6.42 minutes at the main and satellite sites, respectively.

Across all evaluated task categories, satellites generally claimed tasks slightly faster, suggesting reduced bottlenecks and improved POD responsiveness, although the greater clinical support demands at the main campus may also have contributed to this difference. The IQRs were also generally smaller at the satellite sites, indicating that task claim times were more consistent than at the main campus.

- Task‐completion times

Task completion times improved substantially following implementation of the shared‐duty model (Table 3). WCCs exhibited stable and comparable performance across both periods and sites, with consistently narrow IQRs, reflecting an already mature and well‐standardized workflow for WCCs.

**TABLE 3:** The median, and IQR of task completion time at the main and satellite hospitals, with corresponding main‐to‐satellite ratios.

	Before			After		
Task	Main (Minutes)	Satellite (Minutes)	Ratio	Main (Minutes)	Satellite (Minutes)	Ratio
OSPR	45.02/110.55	72.82/95.35	0.62/1.16	32.24/30.49	39.07/35.38	0.83/0.86
RVTC	57.38/67.28	66.67/93.55	0.86/0.72	28.76/33.76	28.65/31.25	1.00/1.08
WCC	5.83/7.00	5.68/6.88	1.03/1.02	5.95/7.05	6.87/7.60	0.87/0.93
CC	46.48/74.37	66.90/103.25	0.69/0.72	9.27/15.73	8.98/15.07	1.03/1.04
Support	120.70/196.62	54.86/112.69	2.20/1.74	18.83/85.43	20.93/63.84	0.90/1.34

OSPR completion times decreased at both locations, with median times declining from 45.02 minutes (main) and 72.82 minutes (satellite) pre‐change to more streamlined and consistent values of 32.24 (main) and 30.49 minutes (satellite) post‐change. RVTC completion times followed a similar pattern, improving from 57.38 and 66.67 minutes before restructuring to 28.76 and 28.65 minutes after implementation. In addition to lower median completion times, the IQRs were generally smaller after implementation, indicating more consistent completion times.

For CC tasks, the improvement was more pronounced: median times decreased from 46.48 minutes (main) and 66.90 minutes (satellite) pre‐change to just 9.27 and 8.98 minutes post‐change—more than a fivefold acceleration at both locations. The corresponding reduction in IQR indicated that completion times also became more tightly clustered.

The most dramatic gains were seen in support tasks. Before restructuring, completion times at the main campus were more than twice those at the satellites (120.70 vs. 54.86 minutes). After the transition, support‐task durations became both markedly shorter and more uniform across sites (18.83 vs. 20.93 minutes), reflecting a substantial improvement in workload distribution and responsiveness.

Overall, the restructuring produced faster, more consistent performance across all major task categories, with the most significant improvements observed for high‐variability support activities.

### 3.4 Safety outcomes

- RO‐ILS events

A total of 171 RO‐ILS events were recorded in the interval before the intervention, of which 53 (30.99% ± 3.54%) were dosimetry‐related. After restructuring, 258 events were reported in the 21‐month interval, including 69 (26.74% ± 2.76%) dosimetry‐related events. A departmental RO‐ILS reporting incentive program was introduced in January 2025. Following introduction of the incentive program, the average monthly number of reported RO‐ILS events increased from 8.49 to 18.86. This represented a numerical decrease of 4.25 percentage points in dosimetry‐related events, although the difference was not statistically significant (*p* = 0.339). At the organizational level, the distribution of dosimetry‐related RO‐ILS event categories shifted following restructuring. Operational/Process Improvement reports increased from 54.72% to 66.67%, whereas Unsafe Condition reports decreased from 13.21% to 5.80%. Near‐miss reports decreased slightly (24.53% vs. 21.74%), Therapeutic Radiation Incident reports remained uncommon (1.89% vs. 1.45%), and Other Safety Incident reports decreased modestly (5.66% vs. 4.35%). These descriptive changes may reflect evolving reporting patterns; however, causality cannot be established because the analyses included all dosimetry‐related RO‐ILS events rather than events specifically attributable to the physics QCL program.

- WCC performance and charge capture

WCC performance improved substantially after the intervention. Before restructuring, PODs completed 12,463 of 12,754 required WCCs (97.7%). Following restructuring, completion increased to 12,582 of 12,593 (99.9%), reducing missed checks to 0.1%.

Charge‐capture accuracy also improved. In the pre‐intervention period, 11,770 of 12,245 eligible WCC charges were processed (96.1%). After restructuring, capture increased to 12,079 of 12,148 (99.4%), an absolute improvement of 3.3%. Taken together, these results demonstrate improved adherence to ASTRO WCC guidelines and more consistent, accurate billing.

### 3.5 Microsoft teams engagement

Daily participation in POD‐related Microsoft Teams channels remained consistently high over a 90‐day assessment period (July 15–October 12, 2025). The average number of active users per day was 10.7 ± 1.9, indicating broad and sustained engagement across all locations. Communication volume was similarly robust: mean daily posts/replies, mentions, and reactions were 53.8 ± 22.4, 20.3 ± 9.3, and 5.6 ± 3.8, respectively. When normalized by active users, participants averaged 5.1 messages, 1.9 mentions, and 0.5 reactions per user per day. Together, these data demonstrate a consistently high level of cross‐site communication within the shared‐duty POD model.

### 3.6 Staff feedback

Surveys were distributed to 20 physicists and 10 dosimetrists, with item‐level response counts ranging from 17–20 and 8–10, respectively. Across both groups, respondents reported broad improvements following POD restructuring.

Among physicists, most indicated clearer task expectations, more equitable workload distribution, and improved day‐to‐day manageability. Notably, 76% reported better workload balance between main and satellite sites, and 80%–90% agreed that the RadOncStatusBoard and Microsoft Teams improved awareness of daily responsibilities. Approximately 85% rated the POD model as efficient, and more than 80% reported increased overall satisfaction.

Dosimetrists expressed similarly positive perceptions. Between 70%–90% endorsed smoother handoffs with physicists, improved support for OSPRs, and greater efficiency in treatment planning. Both groups reported perceived gains in quality and safety, with most indicating that workflow standardization increased, and the risk of missed tasks decreased. Communication with physicists improved for 70% of dosimetrists, though confidence in escalation pathways showed more variability.

Negative responses were uncommon across all survey items, and no item demonstrated a net negative trend. Overall, findings indicate that POD restructuring enhanced communication, safety, task clarity, and workload balance across the department.

## 4 DISCUSSION

Several centers have implemented electronic whiteboards or integrated notification systems to visualize patient flow and task status, with the goal of reducing communication gaps and improving QA through transparent workflows.^20^ Our approach—combining per–clinic‐day Microsoft Teams channels, a real‐time MOSAIQ‐driven task dashboard (RadOncStatusBoard), and active task claiming within PODs—represents a more interactive and integrated implementation. The system incorporates automated logic to validate WCC eligibility, document approval status, and next‐responsible‐party assignment, thereby preventing missed or incorrect tasks. This tightly coupled framework provides end‐to‐end visibility and accountability, distinguishing it from prior literature, which typically presents only individual components (e.g., whiteboards, asynchronous notifications, or Teams‐based handovers) rather than a unified operational solution.

Although satellite PODs generally worked independently on site, all PODs had access to the same departmental resources and could readily consult colleagues when needed. Therefore, QCL volume was used as a measure of workload distribution, recognizing that it may not capture all qualitative aspects of clinical practice. Kim et al.^8^ described workload allocation using normalized task equivalent (nTE) and equivalent workday (eWD) metrics, which quantify workload and improve workload transparency and equity but do not dynamically redistribute responsibilities in response to day‐to‐day clinical demand. In contrast, our shared‐duty model combines cross‐site collaboration with real‐time visibility through the RadOncStatusBoard dashboard to dynamically rebalance workload across PODs, enabling operational responsiveness to day‐to‐day clinical demand in a multi‐site physics practice.

This evaluation demonstrates that the POD restructuring produced measurable improvements in workload balance, task efficiency, and quality metrics across a large, multi‐hospital radiation oncology department. These findings suggest that formalized responsibilities, standardized workflows, and seamless cross‐coverage can improve the consistency, timeliness, and operational resilience of essential physicist activities.

This study has limitations. As a retrospective, single‐institution analysis, generalizability may be limited, particularly for departments with heterogeneous electronic medical record (EMR) or treatment‐planning environments. Although the pre‐ and post‐implementation observation periods occurred during different seasons, introducing the potential for seasonal bias, task volumes remained highly similar between the two periods (within 5% for all major task categories except less frequent support tasks), suggesting that seasonal variation was unlikely to have materially affected the observed changes. Nevertheless, residual seasonal effects cannot be excluded. Time‐to‐claim and time‐to‐complete metrics, although informative, function as surrogate measures; future investigations should assess downstream outcomes such as error interception rates or near‐miss trends. The increase in total RO‐ILS reports should be interpreted in the context of a departmental reporting incentive program introduced during the post‐intervention period, after which monthly reporting increased substantially. Consequently, the absolute number of reported events may not be directly comparable between study periods and may reflect changes in reporting behavior rather than changes in the underlying occurrence of safety events. Therefore, we focused on the percentage of dosimetry‐related events, which is less likely to be influenced by changes in overall reporting behavior. Additional characterization of event severity and linkage of RO‐ILS events to individual physics QCL tasks could provide further insight into the impact of the restructuring. However, such linkage was not available in the current dataset and therefore could not be evaluated. Longer‐term follow‐up with event‐level linkage to the physics workflow will be needed to determine whether these observed changes are sustained and attributable to the intervention. Survey data were collected shortly after implementation and therefore reflect early user perceptions, which may evolve with greater operational maturity.

Overall, these results demonstrate that organizational redesign can produce efficiency, quality, and safety gains comparable to those achieved through new technologies. While advanced imaging and adaptive planning tools continue to expand, ensuring that foundational physics activities are timely, well‐balanced, and transparent may offer more immediate and widespread impact.

Although site‐exclusive models can limit standardization across institutions, they are often a pragmatic response to heterogeneous data, workflows, and governance structures rather than a preferred long‐term organizational strategy. Our findings suggest that as these foundational elements become more standardized, shared operational models such as the POD framework may become increasingly feasible, particularly in healthcare systems undergoing consolidation. During these transitions, site‐specific operational models may serve as an important transitional strategy, accommodating heterogeneous clinical workflows, documentation practices, and data infrastructure until greater harmonization is achieved.

Future work should explore integrating automation or decision‐support tools into the POD framework and investigating whether similar restructuring could benefit adjacent domains such as dosimetry or therapy. Multi‐institutional studies will be essential to evaluate broader applicability and long‐term effects on patient safety, operational performance, and staff well‐being.

## 5 CONCLUSIONS

Restructuring the POD model—supported by a real‐time treatment, QCL, and WCC status board integrated with Microsoft Teams—produced clear, measurable improvements in efficiency, task accountability, and operational consistency across a multi‐hospital radiation oncology network. The redesigned workflow significantly reduced claim and completion times for key safety tasks, improved workload balance between the main and satellite sites, and achieved near‐perfect performance in WCC completion and charge capture. These results demonstrate that intentional workflow design, enabled by real‐time task visibility and cross‐site collaboration, can improve the reliability, standardization, and efficiency of essential clinical physics activities. By illustrating that organizational restructuring can yield meaningful clinical and financial benefits, this work underscores the value of pairing technological tools with thoughtful operational innovation to strengthen the quality and resilience of radiation oncology practice.

## AUTHOR CONTRIBUTIONS

Guang‐Pei Chen, Douglos E. Prah, Eric S. Paulson designed the study. Guang‐Pei Chen collected the data and performed the analysis. Guang‐Pei Chen and Eric S. Paulson contributed to manuscript writing, and all authors approved the final version.

## FUNDING INFORMATION

This research did not receive any specific grant from funding agencies in the public, commercial, or not‐for‐profit sectors.

## CONFLICT OF INTEREST STATEMENT

The authors declare that there are no conflicts of interest regarding the publication of this paper.

## ETHICS STATEMENT

This study was conducted in accordance with the ethical standards of the institutional research committee. Approval for the study was obtained from the Institutional Review Board (IRB) of Medical College of Wisconsin (Approval No. PRO00022316).

## Data Availability

The data that support the findings of this study are available from the corresponding author upon reasonable request.
